# Thoughts on Fang Filling

**Published:** 1860-06

**Authors:** Geo. Watt


### DENTAL REGISTER OF THE WEST.

VOL. XIII.] JUNE, 1860. [No. 12.

### Original Essays and Communications.

### THOUGHTS ON FANG FILLING.

BY GEO. WATT.

THE first article in the present volume of the Register is headed as this one. In that we endeavored to set forth our views on the subject of fang filling. We have followed the same practice invariably since, and have found no good cause for change. We reported a few cases then, and referred to their condition afterward; and we are now ready to report on them again. Will the reader turn back to the first article, in the July number, and compare the cases, as then reported, with their condition now?

No's. 1, 2 and 3 are all right, and No. 4 was in February; No, 5, slight periostitis of same tooth, early in the winter, which soon terminated in resolution; No's. 6 and 7 all right, and No. 8 was in February. No. 9, as previously stated, was all right till the death of the patient.

Without trying to be systematic, we will refer to a few other cases.

CASE.—Mrs. C., aged thirty, bilio-nervous temperament, health bad—predisposition to phthisis. An upper central incisor badly decayed, pulp dead, and abscess formed. The decay was removed, the canal cleansed, and, by means of cotton wrapped on a proper sized quill broach, creosote was pumped into the canal till, by its escharotic effects, it showed its presence at the outlet of the abscess. The canal and cavity were immediately dried, and the tooth was filled in less than two hours from the commencement of the treatment. The discharge of pus ceased immediately, and the abscess was totally healed in a few days. The canal was not filled. This operation was performed about the middle of May, 1859. In February all was right. There had been no return of the abscess, no periostitis—nothing unpleasant in any respect, though the patient's health continues bad. Does any one think we would have succeeded better by filling the fang?

About two years ago we filled two upper bicuspids for Miss P. These teeth were badly decayed, and very much discolored. The pulps had been long dead. After the removal of the decay, a prolonged use of hypochlorite of soda partially restored their color. The crowns *only* were filled. At the end of a year they were considerably darkened. A few months ago we extracted one of them, and about a month ago, the other. We found, what the patient might have told us, that the hard worded operation of "Risodontrypy" had been performed on both. We have no comments to make, but will simply add that, though we have practiced this method *exclusively* for three years, and *generally* for a much longer period, yet these are the first and only teeth we have extracted after treating them as described in our former article. How many such others have extracted, we have no means of knowing. Having carefully tested this practice for more than *six years*, and found it satisfactory, we expect to continue it.

We have been sometimes instructed, frequently entertained, and oftener amused by the objections offered against the practice.

In the January number of the *Cosmos*, a correspondent, "A," after almost acknowledging that our success has been satisfactory, adds, "If (as in most cases it is) an exceedingly thin layer of dentine is sufficient to protect the pulp from any very important changes on account of gold fillings, why is it that the periosteum should be very seriously affected by gold in the fang of a tooth, with the entire wall of dentine between the gold and the periosteum? Will Dr. Watt tell us?"

This correspondent takes a singular course to obtain information. A person who *sincerely* desires to be informed seldom addresses another by *name*, while withholding his own. Then, he asks through the *Cosmos*, while he should have remembered that we *write for the Register*. But, as we are well acquainted with our friend "A," as well as with his motives, we wave all these considerations.

The exact condition of a tooth without a pulp should be well understood, and borne in mind. If the tooth depended wholly on the pulp for its vitality, it would become a foreign body by the death of the pulp. Its vital connection with the system is sustained through the periosteum. To accomplish this, the periosteum takes on increased vital action, which is evidenced by the fact that the periosteum of the pulpless tooth is usually, if not always, more sensitive than that of the normal one. This increased vitality brings it almost to the point of inflammation. From the nature of the tissue, its circulation is easily disturbed, a very slight cause being often sufficient to bring about the change from its ordinary state to that of inflammation. As to the thin layer protecting the pulp, the tooth is then in its normal condition—possesses both its sources of vitality, and is, therefore, better able to withstand the evils of change of temperature. Besides, the circulation of the pulp differs from that of the periosteum, being far less liable to obstruction from depression of temperature. And, where a gold filling is inserted over a very thin layer of dentine, without the intervention of a non-conductor, the pulp, in most, and probably in all successful cases, protects itself by a deposit of bony matter. The same correspondent suggests the alternate application of a hot and a cold instrument to a gold filling inserted as perfectly as possible in the nerve cavity and crown; and, after intimating that it takes the patient sometime to distinguish the hot from the cold instrument, he concludes "that *dead dentine* is almost entirely a non-conductor of heat or cold."

I suppose no one will claim that dead dentine (or any thing else) is a conductor of *cold;* but his suggested experiment does but little toward showing that it is a non-conductor of *heat.* It may be thus demonstrated that the periosteum has but a slight nervous endowment; but we knew this before. If its nervous endowment was equal to that of the pulp, does A. think we could use our teeth in mastication? But if the periosteum possesses but little feeling in its normal state, does that justify us in torturing it into a state of inflammation? We prefer to avoid any course of practice which tends toward this.

We have been somewhat minute in answering this correspondent of the *Cosmos,* not that we can see any special force in his remarks; but, as the senior editor of the *Cosmos* saw fit to publish, and the *three* editors of the *American Review* to republish them, we thought they might be worthy of attention. A. will excuse our delay in answering. He made the request immediately after we had given public notice that we would let the fang filling hobby rest awhile. And at any rate, we are often far too busy with our own correspondents, to be able to respond promptly to those of other journals, even when directly requested to do so.

But, while many have adopted our practice, and are highly successful with it, there is still one objector that might feel slighted if he is not noticed. We allude to our friend of the *Cosmos.* He does not object to our mode because he has tried it and found it a failure. Indeed, from his own statements, it is probable he has never seen teeth treated in this way. He is too cautious, of course, to try it. "Never go into the water, my boy, till you're a good swimmer," said Patrick's mother; and like all good boys, he has taken her advice.

Did you ever hear of the student who, after listening to a lecture on artificial respiration, ran home and tried its effects on a bellows, and finding that it would blow only while the manipulations were continued, pronounced the whole thing a humbug? Our friend, however, has not followed his example. But instead of subjecting the theory we advanced to a practical test—the only way he could tell any thing definite about it—he began his water-soaking experiments which have been before referred to. By these, he endeavored to establish the fact that the serum would accumulate in the pulp cavities, unless they were filled with gold or its equivalent.

Now, it does not follow that, if water will penetrate the tooth substance, in his experiments, it will do so in a tooth treated as we recommended; for the conditions of the teeth are decidedly different. We suggested before that his white wax plug was not reliable; but he tells us it keeps the water out of a glass vial. Is the wax as likely to fit accurately to all parts of a tooth cavity as it does to the mouth of a vial? There is great difficulty in fitting wax so accurately to a tooth that fluids will not penetrate between them. This we learned, *by experience*, years ago. To accomplish the object, we use "adhesive wax," and, while it is still plastic, coat it with collodion. To gratify our friend, we prepared some teeth in this way, tipping the apices with the same material, and put them in water. We examined one three days afterward, and the others in rotation, the last one being submerged for six weeks; and we found *all* the pulp cavities dry. We re-prepared one of them, having filled the pulp cavity with dry plaster of Paris. After soaking this tooth a week, the plaster was still in powder. Will our friend now recognize us as a fellow "soaker?"

But these experiments throw little or no light on our mode of practice. A few successful cases, without a failure, are of infinitely more value. Though our *soaking* experiments annihilates our friend's favorite views, we preferred to have something *on the question*, or at least relating to it. Accordingly, we tried a number of experiments in the mouths of patients. When the teeth were properly prepared for filling we filled the crowns, and had the patients return in from six to thirty days, when we removed the plugs and found, *in all cases*, the pulp cavities dry. We have recently examined over a dozen cases in this way, finding the result as above. In the past year, we have seen many cases, of long standing, in the same condition.

As to discoloration, we are willing to compare our cases with those of our friend. A few weeks ago we saw a central incisor, in the mouth of an eminent dentist, that was filled some fifteen years ago, after the removal of dead pulp, which retained a good color till about a year ago and is but slightly discolored yet, even though the preparatory treatment was not all it should have been.

But we are not discouraged in regard to our friend. He makes progress. In the August number of the *Cosmos*, he says: "The serum, then, having entered the pulp cavity, it remains there;" but in the March number he maintains that *endosmosis* and *exosmosis* take place through all organic structures, whether dead or living, and therefore, of course, if the *liquor sanyuinis* gets into the pulp cavity, it can get out again. But this is of no consequence. We would, however, remind the reader that the inner surface of the walls of the pulp cavity, when treated as we recommend, is no longer an organic structure.

The reader will doubtless notice that our experiments are *practical*, in the mouth, and that they are of several years' standing and are *successful*; and if they apparently or really contradict the wax and water experiments of our *Cosmos* brother, we can not help it. We are not responsible for any controversy that has arisen on this subject. We freely gave our views, and are content with the reception and treatment they have received. We claim the privilege of speaking as freely about the opinion of others; and we take it.

In the last few months we have met with our professional brethren who have given the practice under consideration a fair trial; and, without exception, they are highly pleased with it.

